# Testosterone Reduces Fear and Causes Drastic Hypomethylation of Arginine Vasopressin Promoter in Medial Extended Amygdala of Male Mice

**DOI:** 10.3389/fnbeh.2019.00033

**Published:** 2019-02-26

**Authors:** Wen Han Tong, Samira Abdulai-Saiku, Ajai Vyas

**Affiliations:** School of Biological Sciences, Nanyang Technological University, Singapore, Singapore

**Keywords:** androgen, testosterone, defensive behaviors, semiochemicals, AVP methylation, predator cues

## Abstract

Testosterone reduces anxiety-like behaviors in rodents and increases exploration of anxiogenic parts of the environment. Effects of testosterone on innate defensive behaviors remain understudied. Here, we demonstrate that exogenous testosterone reduces aversion to cat odor in male mice. This is reflected as increased exploration of area containing cat urine when castrated male mice are supplied with exogenous testosterone. We also report that exogenous testosterone leads to DNA hypomethylation of arginine vasopressin (AVP) promoter in posterodorsal medial amygdala (MePD) and medial bed nucleus of stria terminalis (BNST). Our observations suggest that testosterone acting on AVP system within extended medial amygdala might regulate defensive behaviors in mice.

## Highlights

- Exogenous testosterone reduces innate fear to cat odor in male mice.
- Testosterone reduces AVP methylation in extended medial amygdala.

## Introduction

Approach-avoidance conflict has been routinely used to model anxiety in laboratory tests. This includes elevated plus-maze where approach to a novel area and simultaneous avoidance of open spaces is used to create a test with remarkable construct validity. Testosterone induces anxiolysis in this test as manifested by greater time spent on the open arms in both male mice (Aikey et al., 2002) and male rats (Bitran et al., 1993; Frye and Seliga, 2001). Anxiolytic effect of testosterone can also be observed in tests that do not use exploration to construct approach-avoidance continuum, e.g., defensive burying in rats (Fernández-Guasti and Martínez-Mota, 2005). In contrast to anxiety, innate or unconditioned fear is often modeled in laboratory using tests that do not use approach-avoidance conflict as a building block. Instead innate fear is often measured as uni-dimensional avoidance of a predator, or more often an olfactory cue of the predator (Wallace and Rosen, 2000; Dielenberg and McGregor, 2001; King et al., 2005).

Behavioral ecology of innate defensive behaviors, on the other hand, is intertwined with that of reproductive behaviors. The investment in reproductive behaviors often parallels with taking risks and with disengagement from cues of threats within the environment. Thus, imperatives of sexual behaviors are often in conflict with those of protection from predation (Magnhagen, 1991). Pertinent examples include increased parasitism of male crickets caused by eavesdropping of mating calls by parasitoids (Mangold, 1978; Fowler, 1987; Walker and Wineriter, 1991; Walker, 1993). Similarly, sexual investment in acoustic signals enhances predation by bats in bushcrickets and frogs (Zuk and Kolluru, 1998). *Toxoplasma gondii* infection of rats provides another example of the relationship between defensive behaviors and sexual investment. This protozoan parasite is both venereally transmitted from infected males to females and trophically transmitted from infected rats to cats. Rats infected with *Toxoplasma gondii* produce more sexual pheromones that increase their perceived attractiveness to uninfected females (Kumar et al., 2014). The infection also reduces innate aversion to cat odors, suggesting a parallel between sexual investment and reduction in innate fear (Vyas, 2015).

The inverse relationship between defensive behaviors and reproductive investment suggests that these behaviors converge on a similar set of regulatory neuroendocrine mechanisms. It is also likely that testosterone is a crucial part of mechanisms underlying the balance between fear and reproduction. The role for testosterone is supported by its anxiolytic activity, albeit in behavioral experiments that do not directly test aversion to predator cues (Albert et al., 1986; Aikey et al., 2002). Moreover, testosterone is required for reproductive behavior in males (Bialy and Sachs, 2002). For example, production of male pheromones in rats and mice requires testosterone (Kumar et al., 2014; Vasudevan et al., 2015). Thus, it is possible that testosterone can be used as a reliable neuroendocrine signal of ongoing reproductive investment and can lead to reduced defensive behaviors that usually accompany such episodes. This possibility remains hitherto understudied.

Interestingly, both aversion to predator odors and approach to sexual signals require medial extended amygdala within rodent brain (Meredith, 1998; Newman, 1999; Dielenberg et al., 2001; Gross and Canteras, 2012; Bowen et al., 2014). Medial amygdala contains copious amounts of receptors for testosterone and its metabolites (Commins and Yahr, 1985; Simerly et al., 1990; Hines et al., 1992). Posterodorsal part of the medial amygdala is enriched in neurons containing arginine vasopressin (AVP). This neuronal population is part of extra-hypothalamic AVP system and is characterized by its testosterone-dependence (Mizukami et al., 1983; Wang et al., 1993; Wang and De Vries, 1995; Bialy and Sachs, 2002; Cooke, 2006; Auger et al., 2011; Rood et al., 2013). In rats, testosterone causes DNA hypomethylation within the AVP promoter in this brain region (Auger et al., 2011). AVP neurons within extended medial amygdala are also activated during copulation in male mice (Ho et al., 2010) and appetitive approach of male rats to female pheromones in rats (Hari Dass and Vyas, 2014a). In short, role of medial amygdala in both fear and reproduction, and testosterone-responsive nature of AVP neurons within medial amygdala, suggest that these neurons can reduce defensive behaviors during episodes of reproductive preparedness and high testosterone. A testosterone effect on medial amygdala neurons has been shown in rats. Yet, it remains unclear if testosterone causes hypomethylation of AVP promoter in medial amygdala of mice; if similar effects of testosterone are also seen in homologous bed nucleus of stria terminalis (BNST); and if testosterone reduces defensive behaviors.

In this backdrop, we studied effects of testosterone on innate fear by castrating male mice and supplementing them with either placebo or exogenous testosterone over a chronic time window. We also studied ability of testosterone to induce DNA hypomethylation in promoter of AVP gene in two homologous nuclei of medial extended amygdala, posterodorsal medial amygdala (MePD) and posteromedial BNST.

## Materials and Methods

### Animals

Adult male C57BL/6 mice (7–8 weeks old at start of experiment) were procured from InVivos Pte Ltd. Animals were acclimatized for at least 1 week before start of experiments (12:12 light-dark cycle; lights on at 07:00 h; *ad libitum* food and water). This study was carried out in accordance with the recommendations of Institutional Animal Care and Use Committee of Nanyang Technological University. The protocol was approved by the Institutional Animal Care and Use Committee of Nanyang Technological University.

### Castration and Testosterone Supplementation Experiment

All animals were castrated at the start of the experiment. Surgery was conducted in an aseptic environment under deep anesthesia (2% gaseous isoflurane with pure O_2_). Castration was performed by a medial scrotal incision and bilateral removal of testes and vas deferens. Castrated mice were randomly assigned in two experimental groups: those supplemented with placebo (14 mice) and supplemented with exogenous testosterone (12 mice). A silastic tubing (outer diameter = 0.5 cm; inner diameter = 0.3 cm; length = 1 cm; sealed with wood) containing either testosterone or sham was implanted in each animal subcutaneously at the level of the scapula through a small incision at the nape. Testosterone supplementation group received silastic tubing filled with 1.5 mg of testosterone propionate (Sigma-Aldrich). This treatment is reported to result in circulating testosterone concentration similar to that found in uncastrated males (Nyby, 2008). Placebo mice were implanted with empty silastic tubing.

### Cat Odor Aversion Assay

Aversion to bobcat urine was quantified 14 days after implantation of silastic tubing. Experiment was conducted in a rectangular arena (46 × 9 cm) which was divided into two opposing and identical sections. Male mice were first individually habituated in the testing maze for two consecutive days for 1,200 s in the absence of odor. This was followed by another day of habituation in the presence of a novel odor at one terminus (2 ml of 1:4 dilution of vanilla essence). On the following day, animals were exposed to 1 ml of bobcat urine placed at the same terminus where novel odor was placed on the preceding day (Predator Pee, USA). Bobcat urine was replenished after every trial which lasted for 1,200 s. Aversion to cat odor was quantified by the percentage of time spent in bisect containing bobcat urine, relative to total time spent in vicinity of vanilla odor and cat odor.

### Tissue Processing

Animals were restrained and sacrificed immediately after cat odor aversion behavioral assay. Brains were removed and snap frozen in liquid nitrogen. Brains from a randomly chosen sub-sample of placebo and testosterone group were further probed during subsequent assay (*n* = 4 for placebo and 5 for testosterone). The brains were sectioned at 100 μm using a cryotome (−20°C) and mounted on glass slides. Glass-mounted sections were then micro-dissected to harvest paraventricular nucleus (PVN) of hypothalamus, posterodorsal medial amygdala and BNST. Genomic DNA was extracted using DNeasy Blood and Tissue Kit (Qiagen).

### Methylation Sensitive Restriction Enzyme Assay

The extent of AVP promoter methylation was quantified using the methylation-sensitive restriction enzyme (MSRE) digestion assay coupled with quantitative polymerase chain reaction (qPCR). Equal amount of DNA from each mouse was divided into two tubes: restriction enzyme-treated and sans-enzyme. Methylation-sensitive endonucleases, Hpall (New England Biolabs, Ipswich, MA, USA) and BstUl (Fermentas Inc., Glen Burnie, MD, USA), were used to specifically bind and cleave un-methylated DNA at any CCGG and CGCG sequence respectively. Subsequently, primers flanking AVP promoter regions were used to estimate abundance of un-cleaved DNA using qPCR. Hypermethylation in this assay manifests as lower divergence DNA abundance estimates between enzyme-treated and sans-enzyme samples.

### Statistics

Data was analyzed with GraphPad Prism 7.0 software. Inter-group differences were analyzed using independent sample Student’s *t*-test (*p < 0.050, **p < 0.010, ***p < 0.001, ****p < 0.000; *n* is reported in corresponding figure legends).

## Results

### Exogenous Testosterone Reduced Innate Aversion to Cat Odor

**Figure 1:**
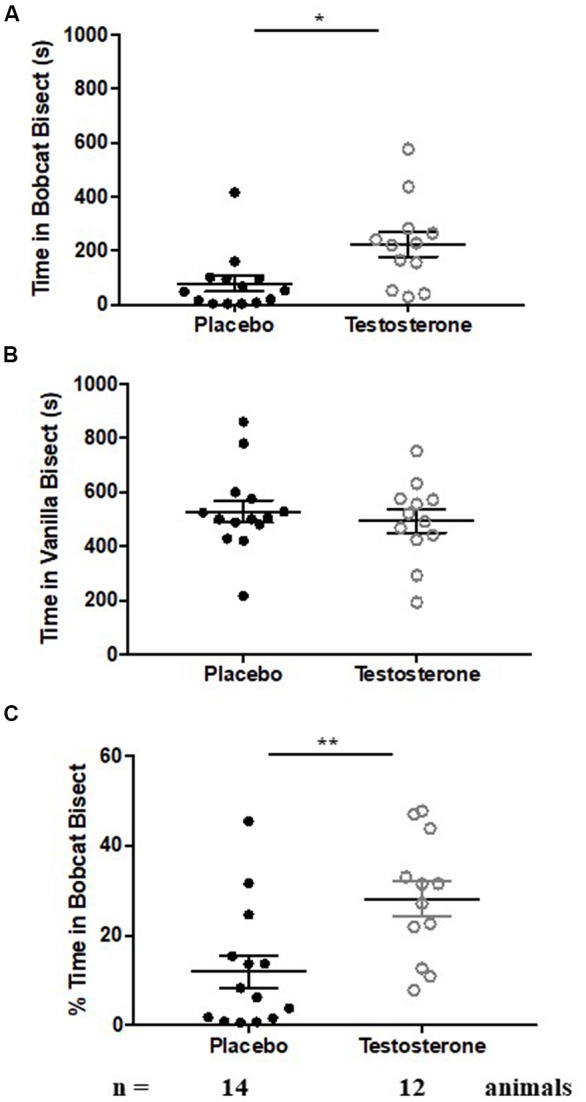
Testosterone supplementation results in reduction in fear to predator odor. Aversion to cat odor in animals chronically supplemented with testosterone (*white circles*) or placebo (*black circles*). Panel **(A)** depicts time spent exploring the bisect containing cat odor. Panel **(B)** depicts time spent exploring the bisect containing vanilla odor. Panel **(C)** depicts percentage time spent exploring cat odor relative to sum of time spent exploring cat and vanilla odor. Bars depict median and inter-quartile range for placebo (*black circles*) and testosterone supplemented (*white circles*) male mice. *p < 0.05, **p < 0.01, independent sample Student’s *t*-test.

Castrated male mice were implanted subcutaneously with testosterone-filled or empty silastic tubing for 14 successive days. Control animals exhibited robust aversion of bobcat urine, as evidenced by lower occupancy in arena bisect containing the predator odor (Figure 1A, placebo group; one-sample *t*-test against chance expectation of 50%; *t*_(13)_ = −18.044, *p* < 0.0001). Control animals did not exhibit aversion to a new non-predator odor on the preceding day (Figure 1B, placebo group; one-sample *t*-test against chance expectation of 50%; *t*_(13)_ = −1.734, *p* = 0.107). Time spent in cat bisect relative to sum of time spent in cat and vanilla bisects also showed statistical significance and robust departure from chance estimate of 50% (Figure 1C, placebo group; one-sample *t*-test against chance expectation of 50%; *t*_(13)_ = −10.487, *p* < 0.0001).

Testosterone supplemented mice retained significant aversion to bobcat odor, shown by reduced occupancy in cat odor bisect (Figure 1A, testosterone group; one-sample *t*-test against chance expectation of 50%; *t*_(11)_ = −8.107, *p* < 0.0001). This aversion was independent of neophobia to non-predator novel odor (Figure 1B, testosterone group; one-sample *t*-test against chance expectation of 50%; *t*_(11)_ = −2.492, *p* = 0.03). Testosterone mice also exhibited significant aversion to cat odor when time spent in cat bisect was normalized to sum of occupancy in cat and vanilla bisects (Figure 1C, testosterone group; one-sample *t*-test against chance expectation of 50%; *t*_(11)_ = −5.552, *p* < 0.0001).

Testosterone supplemented mice exhibited a significant reduction in innate fear during inter-group comparisons (Figure 1A, time spent in cat odor bisect; independent sample-*t*-test: *t*_(24)_ = −2.749, *p* = 0.011; Cohen’s *d* = 1.064). Occupancy in cat bisect for 75% of testosterone supplemented animals was observed to be above the 75th percentile of the control group. The reduction of fear response was present when time spent in cat odor bisect was normalized against the sum of occupancy in vanilla and cat bisects (Figure 1C; independent sample-*t-test*: *t*_(24)_ = −3.009, *p* = 0.006; Cohen’s *d* = 1.134). Inter-group comparison demonstrated no significant difference in time spent exploring the vicinity of vanilla odors (Figure 1B; independent sample-*t-test*: *t*_(24)_ = 0.602, *p* = 0.553; Cohen’s *d* = 0.237; observed power = 0.093 at β = 0.05).

### Testosterone Supplementation Decreased DNA Methylation at Arginine Vasopressin Promoter in Extended Medial Amygdala

**Figure 2:**
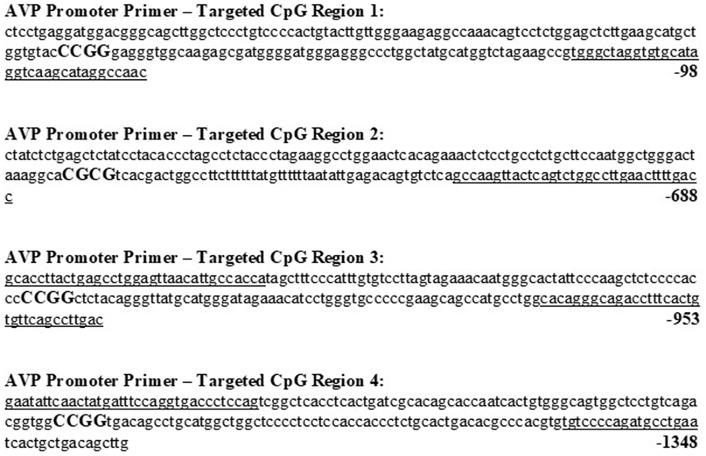
Promoter region of mouse arginine vasopressin (AVP) gene with possible CpG sites, along with primers used to assess methylation levels. Forward and reverse primers for all CpG sites are underlined and CpG sites capitalized and in bold. DNA base sequences and numbering were obtained from Genbank accession no. M88354.1 (1–1,440 base pairs).

NCBI’s Basic Local Alignment Search Tool (BLAST) was used to obtain the mouse AVP promoter region sequence of accession number: M88354.1 (1–1,440 base pairs). Four individual CpG sites were then identified in this DNA sequence using the bioinformatics software EMBOSS Cpgplot^1^, based on abundance of CG content. DNA methylation was experimentally quantified on these CpG sites. Figure 2 depicts targeted CpG sites along with primers used in the DNA methylation assay.

**Figure 3:**
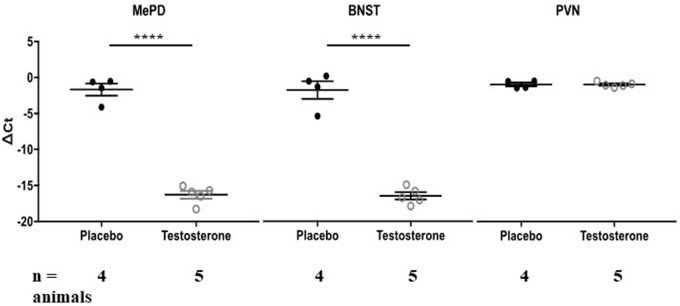
Testosterone supplementation results in hypomethylation of arginine vasopressin (AVP) promoter in posterodorsal medial amygdala (MePD) and bed nucleus of stria terminalis (BNST). DNA methylation status at AVP promoter CpG site 4 was quantified in the MePD, BNST and PVN of all animals chronically supplemented with testosterone (*white circles*) or placebo (*black circles*). Methylation was quantified as ΔCt value (difference in threshold cycle value between samples with or without methylation-specific restriction enzyme treatment). Values that are more negative represent lesser methylation. ****p < 0.000, independent sample Student’s *t*-test.

We quantified methylation status using methylation-specific endonuclease treatment (Hpall and BstUl) and subsequent qPCR. The Hpall enzyme was used to bind three specific CCGG sequences at CpG site 1, 3 and 4. The BstU1 enzyme was used to bind CGCG sequence at CpG site 2. Inter-group comparison revealed that only one CCGG site, located at base 1250 (CpG site 4), displayed significant hypomethylation with the testosterone supplementation treatment in both posterodorsal medial amygdala (MePD) (Figure 3, Tables s 1, 2; independent sample-*t*-test: *t*_(7)_ = 15.163, *p* < 0.0001; Cohen’s *d* = 9.945) and bed nucleus of stria terminalis (BNST) (*t*_(7)_ = 11.908, *p* < 0.0001; Cohen’s *d* = 7.606). Testosterone-treated animals exhibited reduced DNA methylation on this site compared to placebo treatment.

**Table 1:** Raw data of MSRE assay.

	Testosterone	ΔCt	Placebo	ΔCt
MePD	1A	−16.497	2A	−0.530
	1B	−15.679	2B	−1.440
	1C	−18.275	2C	−4.118
	1D	−15.075	2D	−0.609
	1E	−15.910		
BNST	1A	−17.863	2A	−0.509
	1B	−15.812	2B	−1.310
	1C	−16.696	2C	−5.363
	1D	−17.028	2D	0.196
	1E	−14.907		
PVN	1A	−0.459	2A	−0.540
	1B	−1.094	2B	−1.453
	1C	−0.866	2C	−1.390
	1D	−1.376	2D	−0.471
	1E	−1.047		

*Testosterone supplementation results in hypomethylation of arginine vasopressin (AVP) in posterodorsal medial amygdala (MePD) and bed nucleus of stria terminalis (BNST) at AVP promoter CpG site 4. This is observed by the raw ΔCt values between testosterone and placebo groups. There is no difference in ΔCt values in paraventricular nucleus (PVN) which serves as a control to our Methylation Sensitive Restriction Enzyme (MSRE) assay*.

**Table 2:** Statistical analysis of methylation at every CpG site for each brain region.

	CpG	*Z*	*P*-value	Cohen’s *d*	Type II error
MePD	Region 1	−0.490	0.624	0.684	0.162
	Region 2	−0.735	0.462	0.052	0.051
	Region 3	NA	NA	NA	NA
	Region 4	−2.449	0.014	9.945	1.000
BNST	Region 1	−0.289	0.773	0.578	0.129
	Region 2	−0.245	0.806	0.066	0.051
	Region 3	−2.309	0.021	5.524	1.000
	Region 4	−2.449	0.014	7.606	1.000
PVN	Region 1	−1.715	0.086	1.199	0.448
	Region 2	−0.490	0.624	0.352	0.080
	Region 3	0.624	0.221	0.844	0.232
	Region 4	−0.490	0.624	0.012	0.050

*Differences in methylation between placebo-treated and testosterone supplemented male mice for all identified CpG sites. Z-value, p value, effect size and probability of type 2 error for all CpG sites for each brain region*.

We also examined DNA methylation at the corresponding CpG sites in tissues taken from PVN of hypothalamus. Testosterone did not alter methylation status of DNA on the CpG site 4 (Figure 3, Tables s 1; 2; *t*_(7)_ = 0.018, *p* = 0.986; Cohen’s *d* = 0.012; probability of type 2 error = 0.05). Similarly, testosterone did not alter DNA methylation status on CpG sites 1 through 3 (*p* > 0.4). Thus, effects of testosterone on DNA methylation of AVP promoter CpG site 4 were specific to extended medial amygdala.

## Discussion

The observations presented here demonstrate that testosterone reduced defensive behavior in male mice, similar to previously reported effects on anxiety (Bitran et al., 1993; Frye and Seliga, 2001; Aikey et al., 2002). We also show that testosterone leads to epigenetic change in BNST and medial amygdala of male mice in the form of DNA hypomethylation in AVP promoter. This agrees with testosterone-mediated epigenetic changes in the rat’s medial amygdala (Auger et al., 2011).

A variety of animals exhibit greater circulating testosterone when reproductive opportunities are available or when males engage in reproductive behaviors (Harding, 1981; Wood and Newman, 1995; Wood and Coolen, 1997). Supplementation of testosterone-filled cannulas in the medial amygdala of male Syrian hamsters stimulates male sexual and reproductive behaviors (Harding, 1981; Wood and Newman, 1995; Wood and Coolen, 1997). Previous reports have also suggested that increase in plasma testosterone results in increase in aggression (Harding, 1981; Albert et al., 1986). Specifically, plasma testosterone rises in male birds during aggression that is related to reproduction such as mate guarding and territory formation (Wingfield and Ramenofsky, 1985; Wingfield et al., 1990). This suggests that an up-regulation of testosterone increases the propensity of the individual to invest in reproduction. Reproductive episodes are often accompanied by reduced defensive behaviors. Observations in this report suggest that testosterone can mediate reduction in defense in addition to mediating reproductive behaviors. In agreement with our observations, reduced testosterone leads to increased analgesia in rats after exposure to a synthetic chemical reminiscent of fox odor (King et al., 2005). These observations suggest that innate fear and reproductive investment are continually mediated by testosterone and form two ends of an approach-avoidance continuum.

We also show testosterone-mediated epigenetic modification of AVP in posterodorsal medial amygdala (MePD) and BNST. These two nuclei are homologous components of extended medial amygdala (Newman, 1999). AVP produced within the medial extended amygdala is positioned as a prime target to mediate inverse relationship between defense and reproduction. Exposing male rats to estrus females or to copulation increases number of medial amygdala AVP neurons colabeled with Fos, an immediate early gene marker of recent neuronal activity (Hari Dass and Vyas, 2014a). Similarly, the same neuronal population shows increased Fos colabeling during experimental treatment that reduces defensive behaviors, i.e., *Toxoplasma gondii* infection in rats (Hari Dass and Vyas, 2014b). Similarly, inter-individual variation in defensive behavior of male rats can be related to medial amygdala AVP (Bowen et al., 2014). Additionally, both the BNST and MePD contain steroid hormone receptors and thus respond to gonadal steroid hormones and their metabolites (Zhou et al., 1994; Tsukahara et al., 2011). Our data is consistent with these aforementioned studies and suggest a role for the medial extended amygdala in regulation of defensive behaviors during reproductive episodes in male rodents.

Testosterone has been demonstrated to act on steroid receptors and then alter the expression of AVP in MePD (Wang et al., 1993; Wang and Vries, 1995) and BNST of the male rat brain (Wang et al., 1993; Zhou et al., 1994; Gabor et al., 2012). AVP neurons within MePD (MePD-AVP neurons) are responsive to testosterone and its metabolites (Mizukami et al., 1983; Wang et al., 1993; Wang and De Vries, 1995; Bialy and Sachs, 2002; Cooke, 2006; Auger et al., 2011; Rood et al., 2013). This responsiveness takes form of DNA hypomethylation in the AVP promoter (Auger et al., 2011). This epigenetic change provides medial amygdala further information about internal metabolic status and resources needed for investment in sexual pursuits. Thus, testosterone may possibly act as an arbitrator for the trade-off between current and residual reproduction in male mice via the MePD-AVP system. It should nonetheless be noted that the present study does not explicitly quantify the trade-off between reproduction and defense, with this being a difficult task in a laboratory environment. The trade-off between defensive and reproductive behaviors might be mediated by extrinsic ecological factors. For example, testosterone is an immune-suppressant and might explain higher parasite burden of males in natural conditions (Roberts et al., 2004; Muehlenbein and Watts, 2010; Martínez-Guijosa et al., 2015). This can reduce lifespan and thus create a pressure on life history for earlier reproduction. Similarly, reproductive behaviors entail reducing fear and taking risks when males create sexual advertisements or engage in mate searching (Stearns, 1989; Magnhagen, 1991; Grostal and Dicke, 1999). This can increase mortality through predation. Both possibilities require extrinsic ecological factors like parasitism or predation to materialize the trade-off. We have been unable to quantify these in the laboratory model.

Observations in this report present interesting parallels with a naturally occurring perturbation models for diminished defensive behaviors. Male rats chronically infected with the parasitic protozoan *Toxoplasma gondii* display an atypical reduction in innate fear to cat odors (Vyas et al., 2007). This infection creates AVP hypomethylation within medial amygdala while not affecting the PVN (Hari Dass and Vyas, 2014b). The reduction in fear by *Toxoplasma gondii* can be blocked by raising animals on a methyl-donor diet, which also creates AVP hyper-methylation in medial amygdala. This suggests that DNA methylation during the infection is causal to loss of fear. This systemic experiment is supported by a region-specific perturbation. The enzyme DNA methyltransferase is required for methylation of DNA bases. This enzyme can be blocked by RG-108. Prior experiments show that when RG-108 is specifically administered within medial amygdala through a cannula, it results in loss of fear and hypomethylation of AVP (Hari Dass and Vyas, 2014b). Thus, molecular events in *Toxoplasma gondii* perturbation model show remarkable congruence with experimental effects on fear and medial extended amygdala AVP obtained through exogenous testosterone. This further supports the possibility that testosterone, acting through medial amygdala AVP, is a likely mediator of reduced defense.

In conclusion, our data shows that testosterone supplementation leads to reduction of fear response to predator odor. We also provide data that implicates testosterone or its metabolites in altering the methylation state of AVP promoter within the medial extended amygdala. We argue that testosterone increase indicates an intention to mate and this shifts the balance between reproduction and defensive behaviors.

## Data Availability

All datasets generated for this study are included in the manuscript.

## Author Contributions

WT carried out the experiments, performed the data analysis and prepared the manuscript. SA-S carried out the experiments. AV performed the data analysis, conceived the idea, supervised the research and prepared the manuscript. All authors approved the final manuscript.

## Conflict of Interest Statement

The authors declare that the research was conducted in the absence of any commercial or financial relationships that could be construed as a potential conflict of interest.

## Footnotes

^1^http://www.ebi.ac.uk/Tools/seqstats/emboss_cpgplot/

## References

[B1] Aikey J. L. Nyby J. G. Anmuth D. M. James P. J. (2002). Testosterone rapidly reduces anxiety in male house mice (*Mus musculus*). Horm. Behav. 42, 448–460. 10.1006/hbeh.2002.1838 12488111

[B2] Albert D. Walsh M. Gorzalka B. Siemens Y. Louie H. (1986). Testosterone removal in rats results in a decrease in social aggression and a loss of social dominance. Physiol. Behav. 36, 401–407. 10.1016/0031-9384(86)90305-7 3703968

[B3] Auger C. J. Coss D. Auger A. P. Forbes-Lorman R. M. (2011). Epigenetic control of vasopressin expression is maintained by steroid hormones in the adult male rat brain. Proc. Natl. Acad. Sci. U S A 108, 4242–4247. 10.1073/pnas.1100314108 21368111 PMC3053981

[B4] Bialy M. Sachs B. D. (2002). Androgen implants in medial amygdala briefly maintain noncontact erection in castrated male rats. Horm. Behav. 42, 345–355. 10.1006/hbeh.2002.1821 12460594

[B5] Bitran D. Kellogg C. K. Hilvers R. J. (1993). Treatment with an anabolic-androgenic steroid affects anxiety-related behavior and alters the sensitivity of cortical GABAA receptors in the rat. Horm. Behav. 27, 568–583. 10.1006/hbeh.1993.1041 8294123

[B6] Bowen M. T. Dass S. A. Booth J. Suraev A. Vyas A. McGregor I. S. (2014). Active coping toward predatory stress is associated with lower corticosterone and progesterone plasma levels and decreased methylation in the medial amygdala vasopressin system. Horm. Behav. 66, 561–566. 10.1016/j.yhbeh.2014.08.004 25127982

[B7] Commins D. Yahr P. (1985). Autoradiographic localization of estrogen androgen receptors in the sexually dimorphic area and other regions of the gerbil brain. J. Comp. Neurol. 231, 473–489. 10.1002/cne.902310406 3968250

[B8] Cooke B. M. (2006). Steroid-dependent plasticity in the medial amygdala. Neuroscience 138, 997–1005. 10.1016/j.neuroscience.2005.06.018 16330154

[B10] Dielenberg R. A. Hunt G. E. McGregor I. S. (2001). “When a rat smells a cat”: the distribution of Fos immunoreactivity in rat brain following exposure to a predatory odor. Neuroscience 104, 1085–1097. 10.1016/s0306-4522(01)00150-6 11457592

[B9] Dielenberg R. A. McGregor I. S. (2001). Defensive behavior in rats towards predatory odors: a review. Neurosci. Biobehav. Rev. 25, 597–609. 10.1016/s0149-7634(01)00044-6 11801285

[B11] Fernández-Guasti A. Martínez-Mota L. (2005). Anxiolytic-like actions of testosterone in the burying behavior test: role of androgen and GABA-benzodiazepine receptors. Psychoneuroendocrinology 30, 762–770. 10.1016/j.psyneuen.2005.03.006 15919582

[B12] Fowler H. G. (1987). Field behavior of Euphasiopteryx depleta (Diptera: Tachinidae): phonotactically orienting parasitoids of mole crickets (Orthoptera: Gryllotalpidae: Scapteriscus). J. N Y Entomol. Soc. 95, 474–480.

[B13] Frye C. A. Seliga A. M. (2001). Testosterone increases analgesia, anxiolysis, and cognitive performance of male rats. Cogn. Affect. Behav. Neurosci. 1, 371–381. 10.3758/cabn.1.4.371 12467088

[B14] Gabor C. S. Phan A. Clipperton-Allen A. E. Kavaliers M. Choleris E. (2012). Interplay of oxytocin, vasopressin, and sex hormones in the regulation of social recognition. Behav. Neurosci. 126, 97–109. 10.1037/a0026464 22141469

[B15] Gross C. T. Canteras N. S. (2012). The many paths to fear. Nat. Rev. Neurosci. 13, 651–658. 10.1038/nrn3301 22850830

[B16] Grostal P. Dicke M. (1999). Direct and indirect cues of predation risk influence behavior and reproduction of prey: a case for acarine interactions. Behav. Ecol. 10, 422–427. 10.1093/beheco/10.4.422

[B17] Harding C. F. (1981). Social modulation of circulating hormone levels in the male. Am. Zool. 21, 223–231. 10.1093/icb/21.1.223

[B18] Hari Dass S. A. Vyas A. (2014a). Copulation or sensory cues from the female augment Fos expression in arginine vasopressin neurons of the posterodorsal medial amygdala of male rats. Front. Zool. 11:42. 10.1186/1742-9994-11-42 24926317 PMC4054915

[B19] Hari Dass S. A. Vyas A. (2014b). *Toxoplasma gondii* infection reduces predator aversion in rats through epigenetic modulation in the host medial amygdala. Mol. Ecol. 23, 6114–6122. 10.1111/mec.12888 25142402

[B20] Hines M. Allen L. S. Gorski R. A. (1992). Sex differences in subregions of the medial nucleus of the amygdala and the bed nucleus of the stria terminalis of the rat. Brain Res. 579, 321–326. 10.1016/0006-8993(92)90068-k 1352729

[B21] Ho J. M. Murray J. H. Demas G. E. Goodson J. L. (2010). Vasopressin cell groups exhibit strongly divergent responses to copulation and male-male interactions in mice. Horm. Behav. 58, 368–377. 10.1016/j.yhbeh.2010.03.021 20382147 PMC4195792

[B22] King J. A. De Oliveira W. L. Patel N. (2005). Deficits in testosterone facilitate enhanced fear response. Psychoneuroendocrinology 30, 333–340. 10.1016/j.psyneuen.2004.09.005 15694113

[B23] Kumar V. Vasudevan A. Soh L. J. Le Min C. Vyas A. Zewail-Foote M.. (2014). Sexual attractiveness in male rats is associated with greater concentration of major urinary proteins. Biol. Reprod. 91:150. 10.1095/biolreprod.114.117903 25359898

[B24] Magnhagen C. (1991). Predation risk as a cost of reproduction. Trends Ecol. Evol. Amst. 6, 183–186. 10.1016/0169-5347(91)90210-o 21232452

[B25] Mangold J. R. (1978). Attraction of Euphasiopteryx ochracea, Corethrella sp. and gryllids to broadcast songs of the southern mole cricket. Fla. Entomol. 61, 57–61. 10.2307/3494638

[B26] Martínez-Guijosa J. Martínez-Carrasco C. López-Olvera J. R. Fernández-Aguilar X. Colom-Cadena A. Cabezón O.. (2015). Male-biased gastrointestinal parasitism in a nearly monomorphic mountain ungulate. Parasit. Vectors 8:165. 10.1186/s13071-015-0774-9 25888900 PMC4408582

[B27] Meredith M. (1998). Vomeronasal, olfactory, hormonal convergence in the brain. Cooperation or coincidence? Ann. N Y Acad. Sci. 855, 349–361. 10.1111/j.1749-6632.1998.tb10593.x 9929627

[B28] Mizukami S. Nishizuka M. Arai Y. (1983). Sexual difference in nuclear volume and its ontogeny in the rat amygdala. Exp. Neurol. 79, 569–575. 10.1016/0014-4886(83)90235-2 6822281

[B29] Muehlenbein M. P. Watts D. P. (2010). The costs of dominance: testosterone, cortisol and intestinal parasites in wild male chimpanzees. Biopsychosoc. Med. 4:21. 10.1186/1751-0759-4-21 21143892 PMC3004803

[B30] Newman S. W. (1999). The medial extended amygdala in male reproductive behavior a node in the mammalian social behavior network. Ann. N Y Acad. Sci. 877, 242–257. 10.1111/j.1749-6632.1999.tb09271.x 10415653

[B31] Nyby J. G. (2008). Reflexive testosterone release: a model system for studying the nongenomic effects of testosterone upon male behavior. Front. Neuroendocrinol. 29, 199–210. 10.1016/j.yfrne.2007.09.001 17976710 PMC2443938

[B32] Roberts M. L. Buchanan K. L. Evans M. R. (2004). Testing the immunocompetence handicap hypothesis: a review of the evidence. Anim. Behav. 68, 227–239. 10.1016/j.anbehav.2004.05.001

[B33] Rood B. D. Stott R. T. You S. Smith C. J. Woodbury M. E. De Vries G. J. (2013). Site of origin of and sex differences in the vasopressin innervation of the mouse (*Mus musculus*) brain. J. Comp. Neurol. 521, 2321–2358. 10.1002/cne.23288 23239101

[B34] Simerly R. B. Chang C. Muramatsu M. Swanson L. W. (1990). Distribution of androgen and estrogen receptor mRNA-containing cells in the rat brain: an *in situ* hybridization study. J. Comp. Neurol. 294, 76–95. 10.1002/cne.902940107 2324335

[B35] Stearns S. C. (1989). Trade-offs in life-history evolution. Funct. Ecol. 3, 259–268. 10.2307/2389364

[B36] Tsukahara S. Tsuda M. C. Kurihara R. Kato Y. Kuroda Y. Nakata M.. (2011). Effects of aromatase or estrogen receptor gene deletion on masculinization of the principal nucleus of the bed nucleus of the stria terminalis of mice. Neuroendocrinology 94, 137–147. 10.1159/000327541 21525731

[B37] Vasudevan A. Kumar V. Chiang J. Y. Yew J. Y. Cheemadan S. Vyas A. (2015). α2u-globulins mediate manipulation of host attractiveness in *Toxoplasma gondii-Rattus novergicus* association. ISME J. 9, 2112–2115. 10.1038/ismej.2015.33 25853804 PMC4542039

[B38] Vyas A. (2015). Mechanisms of host behavioral change in *Toxoplasma gondii* rodent association. PLoS Pathog. 11:e1004935. 10.1371/journal.ppat.1004935 26203656 PMC4512725

[B39] Vyas A. Kim S. K. Giacomini N. Boothroyd J. C. Sapolsky R. M. (2007). Behavioral changes induced by Toxoplasma infection of rodents are highly specific to aversion of cat odors. Proc. Natl. Acad. Sci. U S A 104, 6442–6447. 10.1073/pnas.0608310104 17404235 PMC1851063

[B41] Walker T. J. (1993). Phonotaxis in femaleOrmia ochracea (Diptera: Tachinidae), a parasitoid of field crickets. J. Insect Behav. 6, 389–410. 10.1007/bf01048119

[B40] Walker T. J. Wineriter S. A. (1991). Hosts of a phonotactic parasitoid and levels of parasitism (Diptera: Tachinidae: Ormia ochracea). Fla. Entomol. 74, 554–559. 10.2307/3495408

[B42] Wallace K. J. Rosen J. B. (2000). Predator odor as an unconditioned fear stimulus in rats: elicitation of freezing by trimethylthiazoline, a component of fox feces. Behav. Neurosci. 114, 912–922. 10.1037//0735-7044.114.5.912 11085605

[B45] Wang Z. Bullock N. A. De Vries G. J. (1993). Sexual differentiation of vasopressin projections of the bed nucleus of the stria terminals and medial amygdaloid nucleus in rats. Endocrinology 132, 2299–2306. 10.1210/endo.132.6.8504734 8504734

[B43] Wang Z. De Vries G. J. (1995). Androgen and estrogen effects on vasopressin messenger RNA expression in the medial amygdaloid nucleus in male and female rats. J. Neuroendocrinol. 7, 827–831. 10.1111/j.1365-2826.1995.tb00722.x 8748118

[B44] Wang Z. Vries G. J. (1995). Androgen and estrogen effects on vasopressin messenger RNA expression in the medial amygdaloid nucleus in male and female rats. J. Neuroendocrinol. 7, 827–831. 10.1111/j.1365-2826.1995.tb00722.x 8748118

[B48] Wingfield J. C. Hegner R. E. Dufty A. M. Jr. Ball G. F. (1990). The “challenge hypothesis”: theoretical implications for patterns of testosterone secretion, mating systems, and breeding strategies. Am. Nat. 136, 829–846. 10.1086/285134

[B47] Wingfield J. Ramenofsky M. (1985). “Testosterone and aggressive behaviour during the reproductive cycle of male birds,” in Neurobiology: Current Comparative Approaches, eds Giles R. Balthazart J. (Berlin: Springer-Verlag), 92–104.

[B49] Wood R. I. Coolen L. M. (1997). Integration of chemosensory and hormonal cues is essential for sexual behaviour in the male Syrian hamster: role of the medial amygdaloid nucleus. Neuroscience 78, 1027–1035. 10.1016/s0306-4522(96)00629-x 9174071

[B50] Wood R. I. Newman S. W. (1995). Integration of chemosensory and hormonal cues is essential for mating in the male Syrian hamster. J. Neurosci. 15, 7261–7269. 10.1523/JNEUROSCI.15-11-07261.1995 7472480 PMC6578098

[B51] Zhou L. Blaustein J. D. De Vries G. J. (1994). Distribution of androgen receptor immunoreactivity in vasopressin-and oxytocin-immunoreactive neurons in the male rat brain. Endocrinology 134, 2622–2627. 10.1210/en.134.6.2622 8194487

[B52] Zuk M. Kolluru G. R. (1998). Exploitation of sexual signals by predators and parasitoids. Q. Rev. Biol. 73, 415–438. 10.1086/420412

